# Hyperlipidaemia and IFNgamma/TNFalpha Synergism are associated with cholesterol crystal formation in Endothelial cells partly through modulation of Lysosomal pH and Cholesterol homeostasis

**DOI:** 10.1016/j.ebiom.2020.102876

**Published:** 2020-07-06

**Authors:** Yvonne Baumer, Amit K. Dey, Cristhian A. Gutierrez-Huerta, Noor O. Khalil, Yusuke Sekine, Gregory E. Sanda, Jie Zhuang, Ankit Saxena, Erin Stempinski, Youssef A. Elnabawi, Pradeep K. Dagur, Qimin Ng, Heather L. Teague, Andrew Keel, Justin A. Rodante, William A. Boisvert, Lam C. Tsoi, Johann E. Gudjonsson, Christopher K.E. Bleck, Marcus Y. Chen, David A. Bluemke, Joel M. Gelfand, Daniella M. Schwartz, Howard S. Kruth, Tiffany M. Powell-Wiley, Martin P. Playford, Nehal N. Mehta

**Affiliations:** a Section of Inflammation and Cardiometabolic Diseases, National Heart, Lung and Blood Institute, 10 Center Drive, Bethesda, MD 20892, USA; b Social Determinants of Obesity and Cardiovascular Risk Laboratory, National Heart, Lung and Blood Institute, 10 Center Drive, Bethesda, MD 20892, USA; c Center for Molecular Medicine, National Heart Lung and Blood Institute, 10 Center Drive, Bethesda, MD 20892, USA; d Cardiovascular and Cancer Genetics Laboratory, National Heart Lung and Blood Institute, 10 Center Drive, Bethesda, MD 20892, USA; e Flow Cytometry Core, National Heart Lung and Blood Institute, 10 Center Drive, Bethesda, MD 20892, USA; f Electron Microscopy Core Facility, National Heart, Lung, and Blood Institute, 10 Center Drive, Bethesda, MD 20892, USA; g Center for Cardiovascular Research, John A Burns School of Medicine, University of Hawaii, 651 Ilalo Street, Honolulu, HI 96813, USA; h Department of Dermatology, University of Michigan, 1301 E. Catherine Street, Ann Arbor, MI 48109, USA; i Section of Cardiometabolic Diseases, National Heart, Lung, and Blood Institute, 10 Center Drive, Bethesda, MD 20892, USA; j Department of Radiology, University of Wisconsin School of Medicine and Public Health, 600 Highland Avenue, Madison, WI 53792, USA; k Department of Dermatology, Hospital of the University of Pennsylvania, 3400 Civic Center Blvd, Philadelphia, PA 19104, USA; l Genetics and Pathogenesis of Allergy Section, National Institute of Allergy and Infectious Diseases, 10 Center Drive, Bethesda, MD 20892, USA; m Section of Experimental Atherosclerosis, National Heart, Lung, and Blood Institute, 10 Center Drive, Bethesda, MD 20892, USA

**Keywords:** Inflammation, Atherosclerosis, Endothelium, Cholesterol crystals, Lipid metabolism, Lysosomal function, Psoriasis

## Abstract

**Background:**

Inflammation plays an important role in the development of cardiovascular disease (CVD). Patients with chronic inflammation diseases have high levels of inflammation and early fatal myocardial infarction due to early, unstable coronary plaques. Cholesterol crystals (CC) play a key role in atherogenesis. However, the underlying mechanisms of endothelial cell (EC)-derived CC formation are not well understood in chronic inflammation.

**Methods:**

We utilized a combination of a mouse psoriasis model (*K14-Rac1V12* mouse model) and human psoriasis patients to study the effect of inflammatory cytokines on CC formation in ECs. Lysosomal pH, alterations in lipid load and inflammatory proteins were evaluated as potential mechanisms linking inflammatory cytokines to CC formation. Coronary CT angiography was performed (n = 224) to characterize potential IFNγ and TNFα synergism on vascular diseases *in vivo*.

**Findings:**

We detected CC presence in the aorta of *K14-Rac1V12* mice on chow diet. IFNγ and TNFα were found to synergistically increase LDL-induced CC formation by almost 2-fold. There was an increase in lysosomal pH accompanied by a 28% loss in pH-dependent lysosomal signal and altered vATPaseV1E1 expression patterns. In parallel, we found that LDL+IFNγ/TNFα treatments increased free cholesterol content within EC and led to a decrease in SOAT-1 expression, an enzyme critically involved cholesterol homeostasis. Finally, the product of IFNγ and TNFα positively associated with early non-calcified coronary burden in patients with psoriasis (n = 224; β = 0.28, p < 0.001).

**Interpretation:**

Our results provide evidence that IFNγ and TNFα accelerate CC formation in endothelial cells in part by altering lysosomal pH and free cholesterol load. These changes promote early atherogenesis and contribute to understanding the burden of CVD in psoriasis.

**Funding:**

Funding was provided by the Intramural Research Program at NIH (NNM) and the National Psoriasis Foundation (NNM and YB).

**Research in context:**

**Evidence before this study**

Chronic inflammatory diseases including psoriasis have an increased risk for cardiovascular disease (CVD) driven mostly by inflammatory-associated vascular diseases. Endothelial cells are capable of cholesterol crystal (CC) production and these CC appear to be important in early atherogenesis. Furthermore, macrophages in psoriatic plaques display an increased presence of CC. Finally, the synergism of two prominent cytokines (IFNγ and TNFα) have been found in the skin and atherosclerotic plaque, but whether these are related to EC production of CC has not been explored.

**Added value of this study**

In this study, we investigated the impact of various cytokines on CC formation, we connect lysosomal pH changes with CC formation, and we present two potentially important regulators for CC formation in chronic inflammatory disease-related CVD. We found that the cytokines IFNγ and TNFα act synergistically to modulate cellular lipid metabolism, lipid homeostasis, and lysosomal function. We also demonstrate that the synergy of these cytokines in human psoriasis associate with early coronary heart disease by CCTA assessed as non-calcified coronary burden.

**Implications of all the available evidence**

Our data strengthen the importance for the need to develop and explore innovative and new treatment strategies especially for the patient populations at higher risk for CVD. Our data provide evidence to focus on targeting dyslipidaemia and treating skin disease to reduce inflammation in psoriasis.

Alt-text: Unlabelled box

## Introduction

Cardiovascular disease (CVD) is the main cause of death worldwide accounting for more than 25% of all death in the US.[1] Inflammation is an important player in CVD development[2] particularly given that CVD is the leading cause of death in chronic inflammatory diseases, including rheumatoid arthritis, lupus erythematosus, and psoriasis.^3^, ^4^, ^5^ Psoriasis, a chronic inflammatory skin disease, affects 2–3% of the world's population and is associated with premature lipid-rich non-calcified coronary plaque and myocardial infarction.^5^, ^6^, ^7^, ^8^

Intact and functional lipid homeostasis is crucial for healthy cells, tissues, and organs, but CVD develops when among other factors, lipid metabolism becomes dysfunctional.[9] Chronic inflammatory-related CVD is at least partially attributed to dysfunctional lipids including a shift to the atherogenic LDL particle phenotype and dysfunctional HDL particle, which is impaired in its ability to support reverse cholesterol transport.[10,11] The impairment in HDL function in several chronic diseases is correlated with prevalent CVD [12] and is further exacerbated by systemic inflammation.[13,14] Several human mutations have also been reported to accelerate CVD morbidity and mortality due to existing genomic and/or functional alterations of lipid metabolism and homeostasis.^15^, ^16^, ^17^ The presence of cholesterol crystals (CC) in atherosclerotic lesions increases over time [18] and has been shown to alter macrophage function and survival, subsequently activating pro-atherosclerotic pathways and accelerating atherogenesis.[19,20] Additionally, CC have been described as potentially an important determinant in determining plaque stability.[21,22] Several cell types have been shown to be capable of active CC production 23, 24, 25, 26, 27, including aortic endothelial cells.[28] Additionally, CC formed within foam cells may be derived from lysosomes.[27,29] In a recent publication, we showed that macrophages, as well as atherosclerotic lesions of psoriatic mice, display increased CC presence.[30] However, the impact of inflammation with dyslipidaemia and its link to CC formation in endothelial cells has not been well characterized.

We utilize human psoriasis as a model of chronic inflammation for the discovery of potential pathways involved in the development of inflammation-related premature atherosclerosis. Additionally, the *K14-Rac1V12* mouse is a reliable model of psoriasis in capturing important features of human psoriatic disease with skin inflammation, systemic inflammation and cardiometabolic dysfunction including accelerated atherosclerosis.[30,31] Additionally, we have previously shown in humans that interferon gamma (IFNγ) and tumor necrosis factor alpha (TNFα) were associated with psoriasis skin disease and also CVD.[32] In the present study, we utilized both mouse and human specimens, and coronary vascular imaging using CCTA to understand the development and association of CC with psoriasis. First, we determined that chronically inflamed *K14-Rac1V12^−/+^* mice on chow diet display similar anatomical features and CC contents as 2-week HFD fed *Ldlr^−/-^* mice, which further substantiated the impact of inflammation on early atherogenesis and CC formation. We found that IFNγ/TNFα synergistically but not individually increased LDL-induced CC formation which was accompanied by an increase in lysosomal pH potentially due to altered vATPaseV1E1 distribution and increased cellular free cholesterol partly due to reduced SOAT-1 expression. Finally, we demonstrate that the product of the plasma levels of IFNγ and TNFα associate with the presence of early coronary artery disease burden in patients with psoriasis.

## Material and Methods

### Human subjects – Ethics Statement

Study approval was obtained from the Institutional Review Board (IRB) at National Heart, Lung and Blood Institute (NHLBI), National Institutes of Health (NIH) in accordance with the principles of Declaration of Helsinki. All guidelines for good clinical practice and those set forth by the NIH Radiation Safety Commission and in the Belmont Report (National Commission for the Protection of Human Subjects of Biomedical and Behavioural Research) were followed. All study participants in the cohort provided written informed consent. All the participants were adequately compensated. Data for all psoriasis patients were obtained under a protocol titled Psoriasis, Atherosclerosis and Cardiometabolic Disease Initiative (13-H-0065), as part of an ongoing observational-cohort study at the National Institutes of Health (NIH) Clinical Centre.

### Coronary Computed Tomography Angiography (CCTA)

All participants underwent coronary computed tomography angiography (CCTA) using 320-detector row CT scanner (Aquilion ONE ViSION, Toshiba, Japan). Scans were performed with EKG gating at 100 or 120 kV, tube current of 100-850 mA adjusted to the patient's body size and a gantry rotation time of 275 ms. Image acquisition characteristics included slice thickness of 0.5 mm with a slice increment of 0.25 mm. Coronary artery characteristics were quantified across all three main coronary arteries using dedicated software QAngio CT (Medis, The Netherlands) by previously described methods. Automated longitudinal contouring of the inner lumen and outer wall was performed, and results were manually adjusted with clear deviations. Total burden (TB) and non-calcified burden (NCB) were calculated by dividing total vessel volume by total vessel length. Lumen attenuation was adaptively corrected on an individual scan basis using gradient filters and intensity values within the coronary artery.

### Measurement of cytokines from human plasma

Human blood samples were collected as described previously.[7] Serum levels of all indicated cytokines were measured using custom U-plex Kits following manufacturer's protocols (Mesoscale, Gaithersburg, MD).

### Mouse model

All mouse experiments were conducted according to IACUC and NIH ASP regulations and guidelines. We utilized heterozygous *K14-Rac1V12^−/+^* mice (skin specific overexpression of constitutively active Rac1^31^), simulating a chronic and systemic inflammatory disease state reported to display cardiometabolic dysfunction [30], and gender-matched littermate controls (LMC) as described previously.[30] Mice were sacrificed, scored, and the aortas harvested and prepared as described previously. [18,30]

### Transmission and scanning electron microscopy

Mouse aortas and HAoEC underwent scanning and transmission electron microscopy (SEM and TEM, respectively). Samples were prepared as described previously.[28,33] SEM imaging was performed using the Hitachi S-3400N1 scanning electron microscope while TEM samples were imaged using a JEM1400 transmission electron microscope both located at the Electron Microscopy Core Facility, NHLBI.

### Substances and materials

All substances/materials with their origin and catalogue numbers or RRID numbers are summarized in the key resource table in the Online Supplement. The following concentrations for cytokines were used: IFNγ at 50 ng/ml, TNFα at 10 ng/ml, IL-1β at 25 ng/ml, IL-6 at 100 ng/ml, IL-8 at 100 ng/ml, and IL-18 at 25 ng/ml. Low density-lipoprotein (LDL) was purchased from Alfa Aesar or Millipore EMD (USA) and used at 500 μg/ml for overnight incubation to simulate hyperlipidaemic conditions. DiI-labelled LDL was used to observe crystal formation by fluorescence microscopy at 100 μg/ml to minimize toxic effects possibly induced by the DiI labelling. Substances used as internal controls or for proof-of-principle experiments were used at the following concentrations: Concanamycin at 100 nM, Chloroquine at 300 nM, and CI-976 at 25 μM. Laboratory derived CC were derived by dissolving cholesterol in 95% ethanol at 60°C followed by a cooling step on ice to allow crystallization. This process was repeated 5-times. Obtained CC were filtered through a Whatman filter paper, grounded using a sterile mortar, and UV light sterilized for 15 min. A sterile stock suspension of CC was prepared in ECGM MV2 and filtered through a 40 μm cell strainer. The filtered and sterile CC suspension was added as a final concentration of 100 μg/ml.

### Human aortic endothelial cell culture

Human aortic endothelial cells (HAoEC) were purchased from PromoCell, Germany. HAoEC were cultured strictly following the manufacturer's recommendations using Endothelial Cell Growth Media MV2 and the PromoCell Split Kit. HAoEC were used up to passage 9. The media was supplemented with the supplement mix provided by the manufacturer, which contains 4% FBS and 2.5 μg/ml free cholesterol.

### Cholesterol crystal formation – Polarize light microscopy (PLM)

Cholesterol crystal formation was observed in 4% PFA/PBS-fixed HAoEC and in 5μm frozen sections of the descending aorta adjacent to the left subclavian artery using the Olympus IX81F-2 microscope with Iplab imaging software. At various points throughout the study, experiments were done in a blinded fashion to ensure accuracy of obtained data. The presence of CC was quantified using ImageJ software. To ensure observed birefringent signal in the *in vitro* setting are indeed derived from LDL, experiments were carried out using DiI-labelled LDL. HAoEC were treated with 4% PFA/PBS for 10 min at room temperature. Subsequently, images were taken in parallel in PL and DiI-red fluorescence emitting channels using the Olympus IX81F-2 microscope with Iplab imaging software allowing the parallel imaging of DiI-positive CC formation by HAoEC.

As an internal positive control for the visualization of CC, laboratory-derived CC were used in all imaging approaches. HAoEC were incubated with 100 mg/ml CC solution for 24 h and afterwards either fixed for PLM or SEM/TEM analysis as described in above sections.

### Detection of lysosomal pH-dependent signal in HAoEC

HAoEC were grown to confluence and treated as indicated. For detection of pH-dependent lysosomal signal, HAoEC were incubated with LysoID Green detection kit (ENZ51034; EnzoLife Sciences, USA) as recommended by the manufacturer and detached from surfaces using the PromoCell split kit. For flow cytometry-based quantification, cells were washed after the incubation and analysed immediately using the LSR Fortessa (BD Bioscience, USA). Data were analysed using FlowJo software (FlowJo9/10, LLC, Ashland, Ore).

For imaging of LysoID, cells were washed after incubation and carefully covered with a glass cover slip and imaged immediately (Echo Revolve Microscope) with the same exposure time throughout each imaging experiment.

### Light Microscopy analysis for monitoring endo-lysosomal acidity

HAoEC were plated in a 35 mm glass bottom dish (MatTek, P35G-1.5–14-C) and allowed to reach 70–80% confluency. Then, cell culture medium was replaced with fresh medium and 1 μg of pCMV-lyso-pHoenix (gift from Christian Rosenmund[34], Addgene #70112; RRID:Addgene_70112), and transfected using Lipofectamine 2000 (Thermo Fisher Scientific, 11668-019) according to the manufacturer's instruction. The next day, the cells were treated with indicated stimuli and cultured for an additional 24 h. LysoTracker Blue DND-22 (Thermo Fisher Scientific, L7525) was added (final 1 μM) 1 h before and washed out by replacing medium just before observation. Fluorescent images were acquired using a Zeiss LSM 780 Confocal Microscope (Zeiss) equipped with a 63 × /1.4 NA oil immersion objective and driven by ZEN software. Excitation lasers and emission ranges for detection were set as follows; LysoTrackerBlue; 405 nm and 415–499 nm, super-ecliptic pHluorin; 488 nm and 499–553 nm.

### Fluorescence microscopy staining of HAoEC

HAoEC were treated as indicated and fixed using 4% PFA/PBS for 10 min at room temperature (RT). All antibodies are shown in the Key Resource Table. Visualization of the actin cytoskeleton, as an indicator for intactness of the endothelial monolayer, was performed by incubation of fixed HAoEC with Phalloidin-Alexa 488 (1 μM in 2% BSA/PBS, Invitrogen USA) for 30 min in the dark at RT. For all other immunofluorescences, fixed HAoEC were permeabilized using 0.1% Triton X-100 in PBS for 5min at RT, washed 3 times with PBS, and blocked by a 1 h incubation with 2%BSA/10% normal goat serum in PBS at RT. Primary antibodies were diluted 1:100 in 2% BSA/PBS and incubated for 24 h at 4°C. At this point, cells were thoroughly washed using PBS 3-times and the secondary cy3-labeled antibody added at a 1:500 dilution in 2% BSA/PBS for 1 h at RT in the dark. For all stainings, a labelling of nuclei was performed using DAPI by adding it to the antibody mix for 5 min in the dark. Cells were than washed with PBS 3 times and mounted with a cover slip using DAKO fluorescence mounting media (S302380-2, DAKO, USA). All fluorescence images were acquired using the Revolve Echo Microscope (Echo, USA).

### Flow cytometry of HAoEC

Content of esterified/neutral and free cholesterol was determined by flow cytometry. HAoEC were treated as indicated, removed from the surfaces using the PromoCell splitkit, and fixed using 1% PFA in flow buffer. Afterwards, cells were labelled with either 25 μg/ml Filipin in 10%FBS/PBS (F9765, Sigma, USA) or HCS LipidTOX Deep Red (1:1000 dilution in PBS, H34477, ThermoFisher Scientific, USA) 30 min at RT in the dark. Cells were thoroughly washed using PBS and resuspended in flow buffer containing 1% PFA. The uptake of LDL was performed in two ways: a) subsequent to cytokine/LDL treatment, by incubating pre-treated HAoEC with 10 μg/ml DiI-LDL for 2 h; or b) 24 h treatment with 100 μg/ml DiI-LDL in presence of indicated cytokines to allow quantification of 24 h lipid uptake and overlay of birefringent CC and DiI-detectable signal. Cells were then removed from the cell culture surface using the PromoCell splitkit and fixed using 1% PFA in flow buffer.

Expression of LAMP1 was determined by flow cytometry. For intracellular staining, HAoEC were de-attached from surfaces using the PromoCell splitkit, fixed and permeabilized using the Fixation/Permeabilization Solution kit (554714, BD, USA) for 5 min at RT, thoroughly washed with the supplied washing buffer and stained with the antibodies in wash buffer for 15 min in the dark on ice. Afterwards cells were washed using flow buffer and fixed. All flow experiments were performed using the LSR Fortessa (BD Bioscience, USA). Data were analysed using FlowJo software (FlowJo, LLC, Ashland, Ore).

### Statistical analysis for *in vitro* and *in vivo* experiments

Statistical comparison between groups was performed using PRISM 8.0 (GraphPad) software. Data are represented as mean ± the standard error. Before every statistical test, normal distribution of each dataset was tested using the D'Agostino/Pearson normality test. Normality for an experiment was assumed only if all treatment datasets of the specific experiment were normally distributed. If one treatment of an experimental set was not normally distributed, all data were treated as not normally distributed. Normally distributed datasets were either evaluated using t-test to compare two variables with each other. To allow for multiple comparisons within normally distributed dataset the RM one-way ANOVA Tukey's multiple comparison test was performed. Nonparametric datasets were analysed using Mann-Whitney U test to compare two variables with each other. For multiple variable comparisons the Friedman ANOVA test with Dunn's multivariable comparison was used when comparing all variables to each other within a group. When comparing all data within an unpaired dataset the Kruskal-Wallis test along with Dunn's multiple comparison test was performed. Spearman correlation was used for correlation of in vitro data which were nonparametric. Statistical significance was evaluated according to statistical tests as stated in each figure legend and established at a p value of p < 0.05. Exact n-numbers for each experiment are given in the figure legends and represent independent repeats of the displayed experiment.

### Statistical analysis (human studies)

Summary statistics were generated and expressed as mean with standard deviation for parametric variables and median with interquartile range for non-parametric continuous variables. Categorical variables were recorded as frequencies and percentages. Normality was evaluated using skewness, kurtosis, and histogram plots. Intergroup differences were assessed using Student's t-test and Mann-Whitney U test for parametric and non-parametric data respectively. Dichotomous variable comparisons were conducted using Pearson's chi-square test. We conducted univariable and multivariable linear regression analyses to evaluate the associations of cytokine synergism with non-calcified coronary artery burden in the psoriasis cohort. The potential confounding variables were determined for all parsimonious models based on purposeful selection and prior published literature. Standardized beta values are reported, which indicate number of standard deviations change in the outcome variable per standard deviation change in the predicting variable. Linearity of the data was assessed for outliers and sensitivity analysis was performed after dropping outliers which, determined by r values, and results were not found to be significantly altered. Normality of regression models was assessed using Shapiro-Wilk test and IQR test with lack of significant p-values resulting in acceptance of null hypothesis for normality. We also checked for homoscedasticity of residuals and found constant residual variance for regression models. Two-tailed p-values were reported throughout all analyses and p-value ≤0.05 was deemed significant. All statistical analyses were performed using STATA 12 (StataCorp., College Station, TX, USA).

## Results

### The presence of CC in the aorta of K14Rac1 murine model of psoriasis

**Fig. 1:**
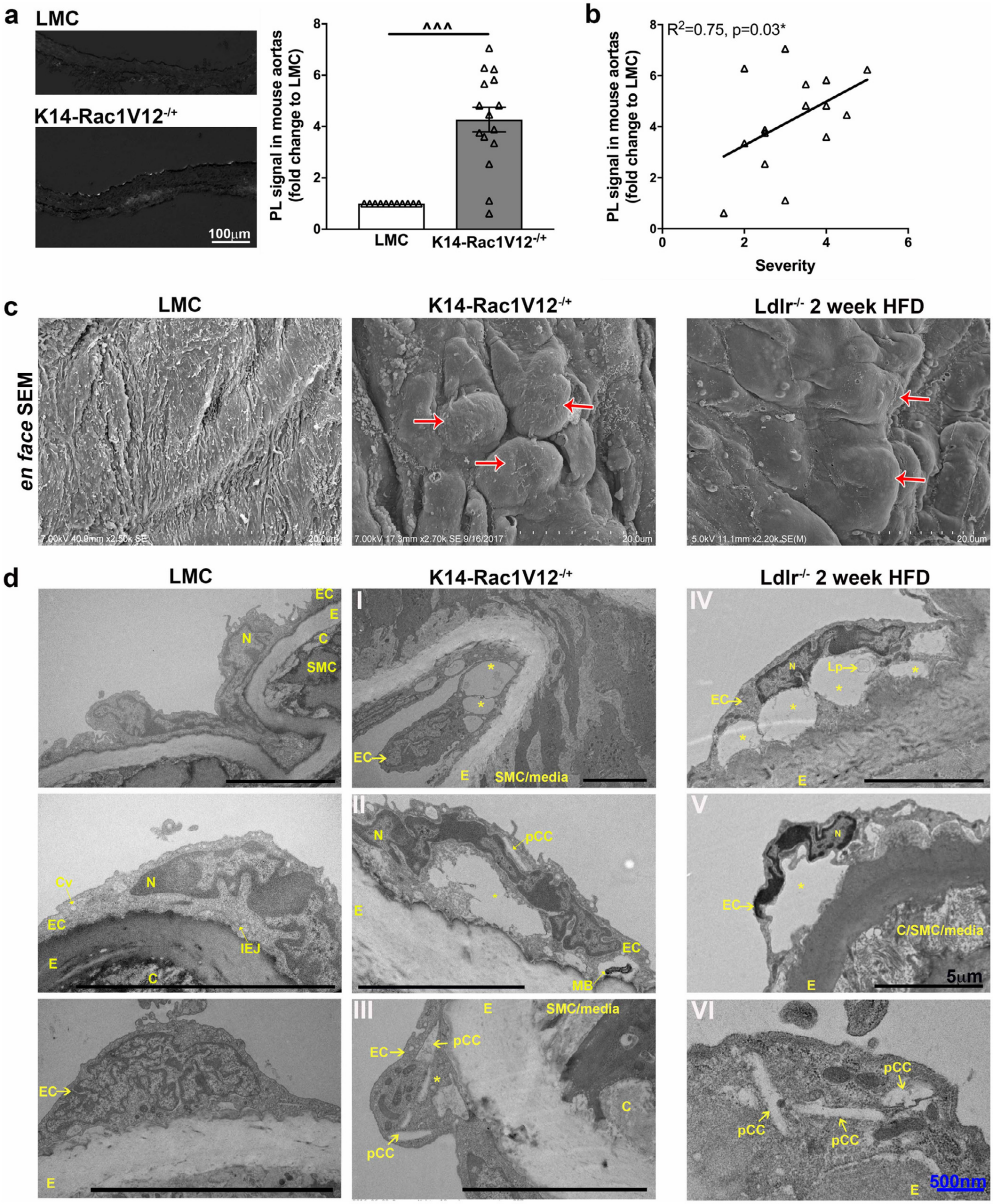
Presence of cholesterol crystals in psoriatic mouse aortas on chow diet. Aortas of *LMC* and *K14-Rac1V12^−/+^* mouse aortas on chow diet were harvested and subjected to PLM and ultrastructural analysis using TEM and SEM. (a) Sections of the aorta were visualized by PLM and the area directly under the endothelium emitting a birefringent signal, representative of CC, quantified (n*LMC/ K14-Rac1V12^−/+^* = 11/15). (b) The data from (a) in *K14-Rac1V12^−/+^* mouse aortas were subjected to Pearson correlation after normality check and revealed a strong positive association of CC presence in the aorta and severity in animals not being fed a HFD. (c) The aortic arch of a subset of these mice (each n = 3) was subjected to SEM. Imaging the surface of the aortas of chronically inflamed *K14-Rac1V12^−/+^* mice revealed the presence of large areas seemingly bulging out. A loss of the smooth surface found in LMC was observed. In comparison with images of *Ldlr^−/−^* mice after 2-week HFD are shown displaying similar features. (d) TEM ultrastructural analysis revealed the presence of subendothelial spaces and clefts within the endothelium of *K14-Rac1V12^−/+^* mice on chow diet, which could not be observed in LMC mice (n = 3). Strikingly similar pathological changes of the aortic wall were observed in *Ldlr^−/−^* mice after 2-week HFD, representative of a very early atherogenesis stage. (red arrow-indicate bulged areas representative for very early stages of atherogenesis; yellow Asterix-subendothelial spaces/’clefts’; black scale bar – 5 μm, blue scale bar – 500 nm; Abbreviations: C-collagen, Cv-caveolae, E-elastin fibrous layer, EC-endothelial cell, IEJ-interendothelial junction, N-nucleus, LMC-littermate control, Lp-lipid particle, pCC-possible CC clefts, SMC/media-smooth muscle cell/tunica media layer)

Previously, we demonstrated that the *K14Rac1V12* mouse model displayed increased levels of pro-inflammatory cytokines and when crossed in an atherosclerosis mouse model the atherosclerotic plaques showed increased presence of CC.[30] In the present study, aortas of *K14-Rac1V12^−/+^* mice on chow diet were examined using PLM to detected birefringent signal, representative for CC. We noted a 4.2-fold increase of CC presence when compared to LMC aortas (Fig. 1a). Interestingly, the content of CC detected in the subendothelial layer of the aorta significantly correlated with the psoriasis severity score determined for mice used in this study (R^2^=0.75, p=0.03; *Pearson correlation*) (Fig. 1b). Examining the aortas by scanning electron microscopy (SEM, Fig. 1c) we determined that the surface of these aortas was uneven and covered in pockets seemingly filled. The phenotype of these aortas resembled *Ldlr^−/−^* mice on HFD.[18,28] When comparing *K14-Rac1V12^−/+^* mouse aortas on chow diet with *Ldlr^−/−^* mouse aortas after 2 weeks of HFD, we observed similar findings (Fig. 1c). Next, we further studied the ultrastructure of these blood vessels using transmission electron microscopy (TEM, Fig. 1d). In LMC aortas, the endothelial layer was found to be in close contact with the underlying layers. However, *K14-Rac1V12^−/+^* mice displayed large clefts in the subendothelial space which in the literature has been attributed to deposited lipids and/or CC [18,28] (Fig. 1d, I-III). Again, we compared the detected phenotype to aortas of *Ldlr^−/−^* mouse aortas after 2 weeks of HFD (Fig. 1d**, IV-VI**), which displayed close similarities to the non-HFD fed *K14-Rac1V12^−/+^* mice.

### Pro-inflammatory cytokines associate with endothelial cell-derived CC formation *in vitro*

**Fig. 2:**
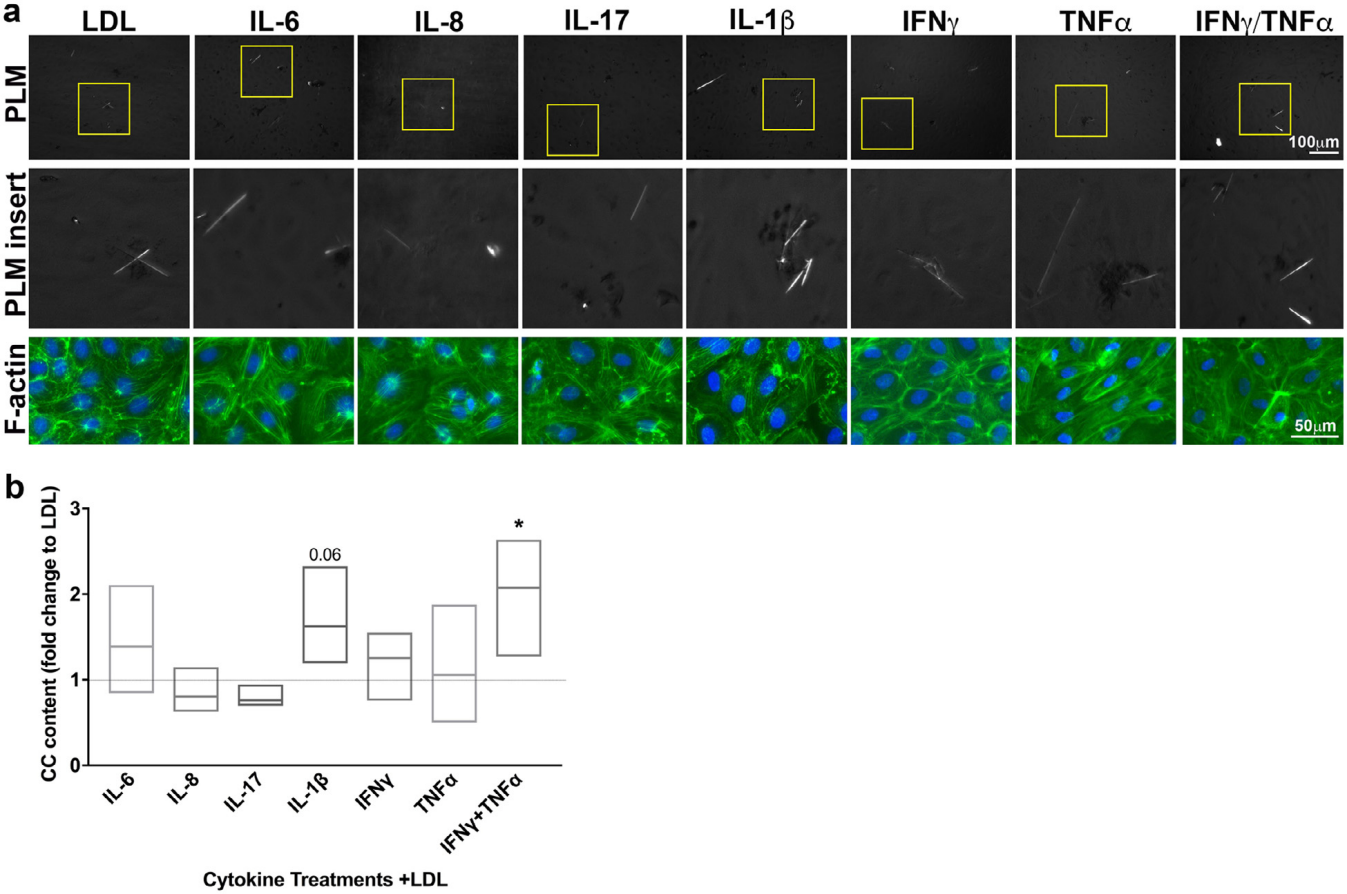
The impact of a pro-inflammatory milieu on LDL-induced CC formation in HAoEC. (a/b) HAoEC were treated with LDL in the presence of various pro-inflammatory cytokines. Subsequently, HAoEC were subjected to PLM to assess CC formation (a representative PLM image for each treatment is shown). Presence of CC was then quantified using ImageJ. (b) (n = 6 each, Kruskal-Wallis/Dunn's multivariable comparison test; * indicates significance to LDL treated HAoEC). To ensure treatments did not result in massive cell death possibly altering CC formation artificially, HAoEC were afterwards labelled with Phalloidin to image the actin cytoskeleton(green) and ensure EC monolayer existed. Nuclei were labelled using DAPI (blue) (n = 3).

The presence of spontaneously occurring CC in *K14-Rac1V12^−/+^* mice strongly suggested that psoriasis-associated inflammatory mediators could induce atherogenesis *in vivo*. A number of pro-inflammatory cytokines are elevated in murine and human psoriasis; this led us to hypothesize that one of these cytokines might directly induce CC production in endothelial cells. The ability of cells to produce CC was to our knowledge first reported in macrophages in 1994.[27] Since then, other cell types like smooth muscle cells and endothelial cells have been demonstrated to be capable to produce CC under hyperlipidaemic conditions.[26,28] Therefore, confluent monolayers of HAoEC were treated with a variety of pro-inflammatory and psoriasis-related cytokines with addition of LDL. The presence of CC was examined by PLM and subsequently quantified (Fig. 2). To ensure that observed CC formation was not a result of a loss of the EC monolayer, cells were subsequently labelled with Phalloidin to visualize the actin cytoskeleton. We found that IL-1β enhanced CC formation when compared to LDL (1.66 ± 0.15-fold change, p = 0.09; Kruskal-Wallis test with Dunn's multivariable comparison test, (Fig. 2b), while IL-6, IL-8, IL-18, IFNγ, or TNFα had no significant effect on CC formation. We additionally tested a possible synergistic effect of IFNγ and TNFα on CC formation due to our prior findings.[32] Combination of both cytokines displayed a significant upregulation of LDL-induced EC CC formation with 1.95 ± 0.23-fold (p-value 0.03, Kruskal-Wallis test with Dunn's multivariable comparison test) when compared to LDL treatment alone (Fig. 2b). In another step verifying that observed CC after LDL+IFNγ/TNFα treatment were derived from LDL, we treated HAoEC with the cytokines individually or in combination in presence of DiI-labelled LDL to overlay the birefringent signal with the DiI-signal and by this confirm that observed birefringent structures are derived from LDL (Supplementary Figure 2).

### IFNγ/TNFα synergism in hyperlipidaemic conditions modulates lysosomal pH

**Fig. 3:**
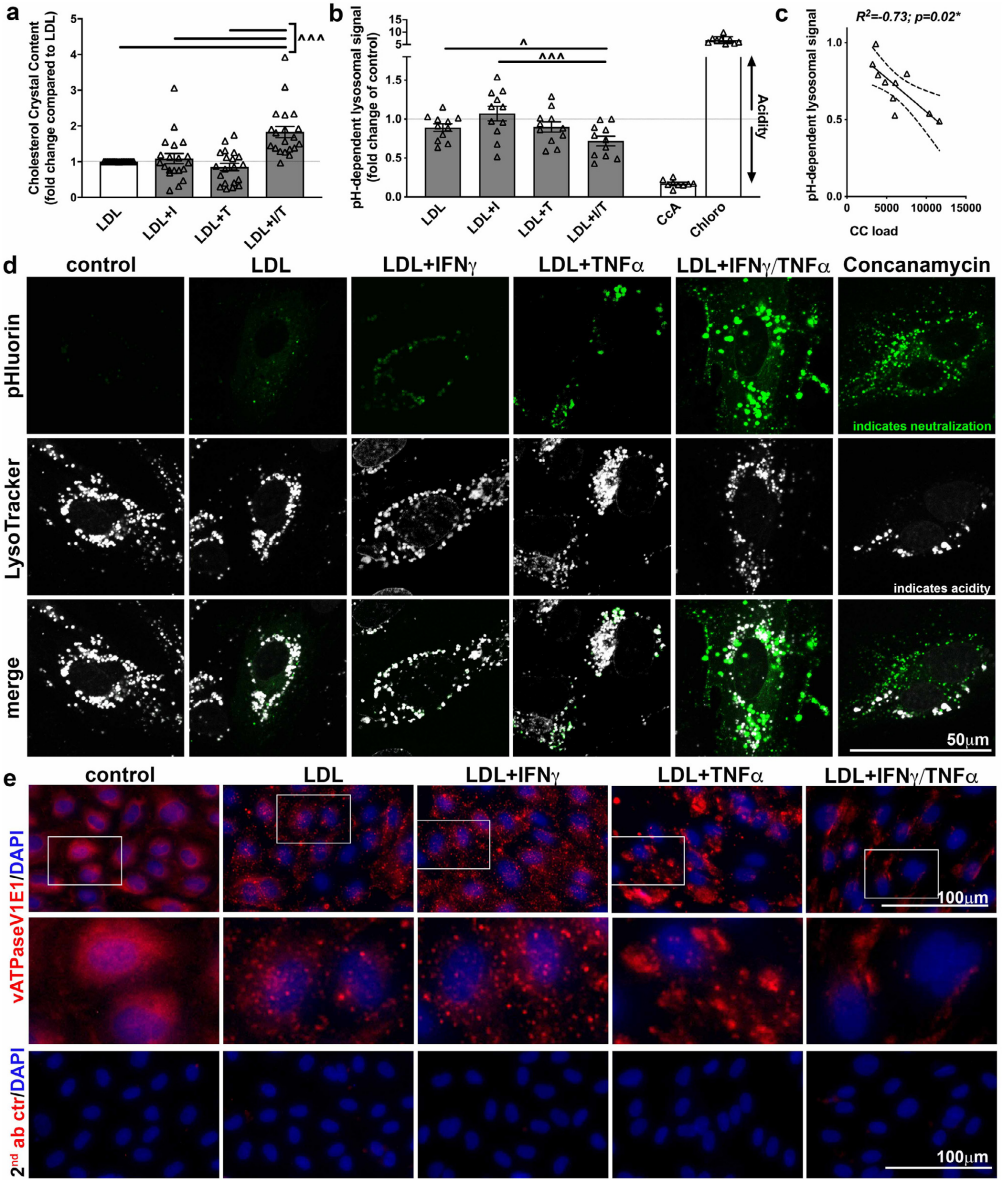
The impact of IFNγ and TNFα synergism on cholesterol crystal formation and endothelial lysosomal pH. Human aortic endothelial cells (HAoEC) were treated as indicated for 24 h. (a) Presence of cholesterol crystals (CC) was observed using PLM. CC-positive area was quantified using ImageJ, and is displayed as a fold change to LDL. Multivariable comparison between all treatment groups (non-parametric data, paired) was performed using a Friedman test with a Dunn's multiple comparison test (n ≥ 10, ^ indicates significance between indicated groups). (b) HAoEC were treated as indicated and subjected to flow cytometry identifying pH-dependent lysosomal signal using LysoID and intracellular staining of LAMP1 in parallel. Afterwards the LysoID signal was normalized to the presence of lysosomes to determine a pH-dependent lysosomal signal (n = 10). Multivariable comparison between all treatment groups (normal distributed data, paired) was performed using a RM one-way AN. OVA test with a Turkey's multiple comparison test (^ indicates significance between indicated groups). Concanamycin (CcA), a vATPase inhibitor (pH increase), and chloroquine, a lysosomal pH decreasing drug, were used as internal assay positive controls. (c) Obtained results from crystal analysis (a) and pH-dependent lysosomal signal (b) from in parallel conducted experiments were subjected to a Spearman correlation. (d) HAoEC were transfected with a construct enabling detection of green fluorescence when present in neutral instead of acidic lysosomes (pHluorin). In parallel, acidic lysosomes were labelled using LysoTracker (white) (n = 3). IFNγ/TNFα+LDL treated cells display an increase in neutral lysosomes while acidic lysosomal compartments decrease in number. (e) HAoEC were treated as indicated for 8h. The V1E1 subunit of the lysosomal vATPase was stained using immunofluorescence. Decreased expression and altered distribution can be detected. All images (including the secondary antibody control) were taken with the same exposure time. (n = 3).

CC in macrophages are formed within lysosomes.[27] Additionally, pH impacts size and amount of CC formed under chemically defined conditions.[35] We therefore aimed to determine if IFNγ and TNFα possibly synergistically alter lysosomal pH and support LDL-induced CC formation. Experiments were carried out in parallel to allow correlation of obtained results (Fig. 3a–c). As in the initial set of experiments, HAoEC were treated with LDL alone or in presence of IFNγ, TNFα, or IFNγ and TNFα together. Subsequent CC quantification and statistical analysis of PLM images revealed that IFNγ and TNFα synergistically and significantly increased LDL-induced presence of CC by 1.8 ± 0.2-fold as compared to single treatment counterparts in this set of experiments (fold changes to LDL: LDL+IFNγ 1.1 ± 0.1, LDL+TNFα 0.9 ± 0.1, or LDL+IFNγ/ TNFα 1.8 ± 0.2). To explore the possibility of altered lysosomal pH under the indicated treatment conditions LysoID, a substance emitting a green fluorescence with its intensity being dependent on lysosomal pH, was assesed with IF and flow cytometry(Supplementary Fig. 3a/b). We found an increase in lysosome presence and size indicating the importance normalizing the detected LysoID signal to intracellular LAMP1 expression (Supplementary Fig. 3c) to determine a pH-dependent lysosomal signal (Fig. 3b). IFNγ and TNFα in the presence of LDL synergistically decreased pH-dependent lysosomal signal by 28 ± 6%, compared to the individual treatments (LDL: 11 ± 5% decrease, LDL+IFNγ unchanged, LDL+TNFα: 10 ± 7% decrease), indicating a change in lysosomal pH. When we correlated the loss of detected pH-dependent lysosomal signal to the detected CC load from the same experiments, we found a strong and significant correlation for these *in vitro* experiments *(R^2^* *=* *-0.73, p* *=* *0.02, Spearman correlation*) (Fig. 3c).

To verify our findings from above and further characterize the potential change in lysosomal pH we utilized a second methodological approach. HAoEC were transfected with the lyso-pHoenix probe, which is exclusively expressed in lysosomes and only emits a green fluorescence if lysosomes display a neutral pH. In contrast to the aforementioned flow cytometry technique used (where a loss in green fluorescence indicated neutralization of lysosomal pH), this microscopy-based technique detects neutral lysosomal pH by emission of green fluorescence. HAoEC were then counter-labelled with LysoTracker (displayed in white) to label all lysosomes with acidic pH (Fig. 3d). Subsequent fluorescence imaging showed increased numbers of neutral lysosomes (green) over acidic lysosomes (white) in LDL+IFNγ/TNFα treated HAoEC when compared to the individual treatments, indicating that IFNγ and TNFα in presence of LDL likely synergistically neutralize lysosomal pH.

Lysosomal pH is partly regulated by vATPases, a highly conserved set of proteins acidifying many intracellular organelles. Dysregulation of vATPase's subunits has been implicated in cancer and neurodegenerative diseases.[36] Utilizing IF (Fig. 3e), we found that vATPaseV1E1 was present in HAoEC in a punctuated pattern throughout the endothelial cells which did not change when cells were treated with LDL or LDL+IFNγ. However, the punctuated pattern of V1E1 was reduced and localized in larger accumulations in LDL+TNFα treatment. Almost no vATPaseV1E1 pattern could be detected when treated with LDL+IFNγ/TNFα, indicating a loss in expression or ‘misrouting’.[37] These changes in vATPaseV1E1 may indeed be causative for lysosomal pH changes and thus warrant further investigation. As a first proof-of-principle experiment, Concanamycin (neutralizes lysosomal pH) was used in the presence of LDL. To our disappointment while pH-dependent lysosomal signal significantly decreased, no significant changes were seen in CC formation (Supplementary Fig. 3d/e).

### IFNγ/TNFα synergism in presence of LDL increases free cholesterol load in HAoEC with concomitant SOAT-1 expression

**Fig. 4:**
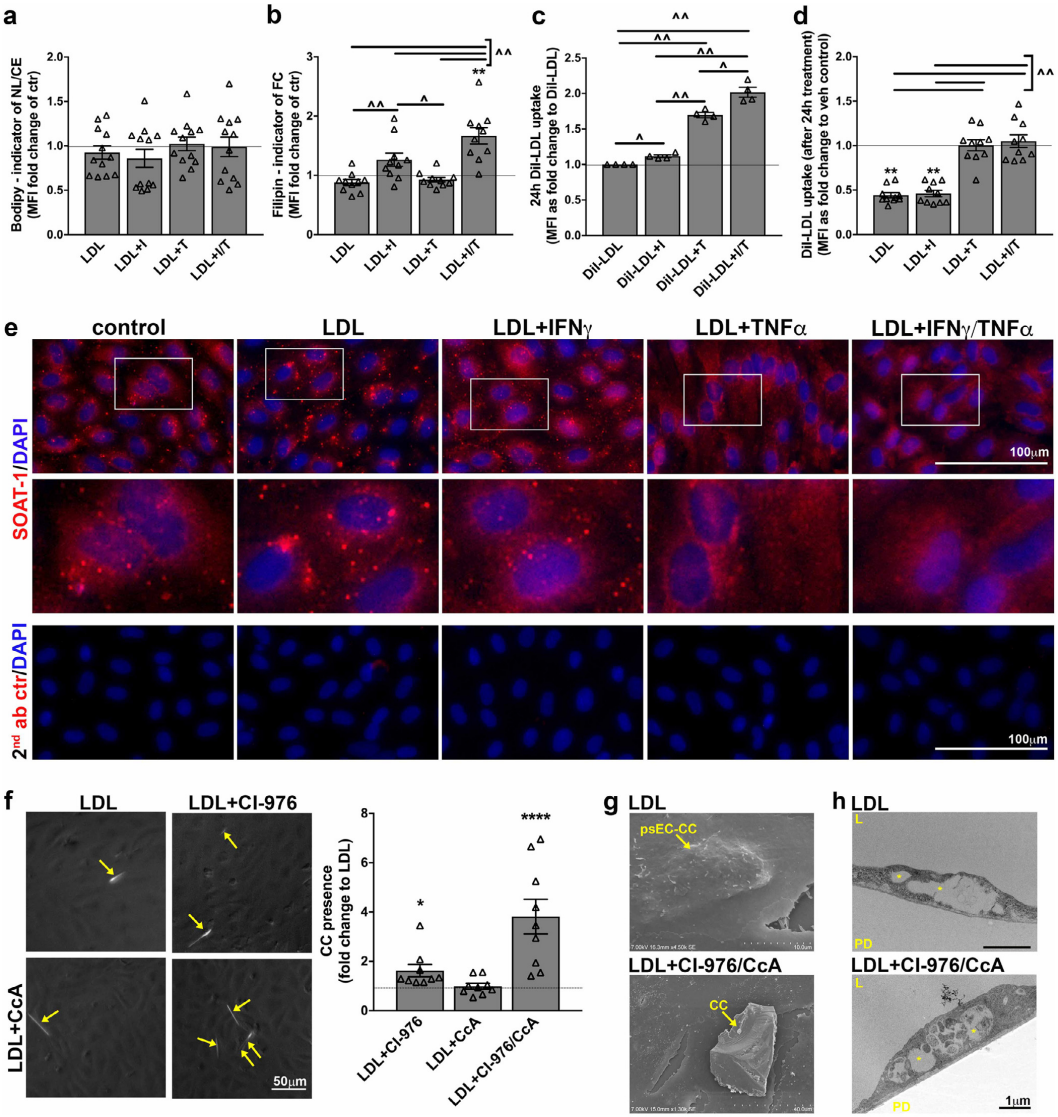
IFNγ and TNFα synergism potentially increases CC presence by increasing free cholesterol load due to altering SOAT-1 expression. Human aortic endothelial cells (HAoEC) were treated with IFNγ, TNFα, and LDL and each possible combination for 24 h and the impact of the presented treatments on neutral lipid/cholesterol ester load (a) and free cholesterol load (b) examined using flow cytometry (n = 10 each). (c) To test overall lipid uptake within 24 h of treatment cells were treated as indicated utilizing DiI-labelled LDL. DiI fluorescence was detected after 24 h using flow cytometry. (n = 4) (d) To assess the LDL-uptake potential of HAoEC after 24 h of indicated treatments and therefore the potential alteration of lipid sensing mechanisms, HAoEC were after completed 24 h indicated treatment incubated for 2 h using DiI-LDL. The presence of DiI-fluorescence is an indicator for the uptake potential of LDL after 24 h of initial indicated treatments was determined by flow cytometry. (n = 10) (Statistics: *significance to vehicle control, ^significance between indicated groups; all treatment groups were statistically compared to each other: ‘c were analysed using Friedman/Dunn's multiple comparison test, while ‘a+b+d’ displayed normal distribution and therefore RM one-way ANOVA/Turkey's multiple comparison test was used) (e) HAoEC were treated as indicated for 8h. SOAT-1, the enzyme catalysing free cholesterol to cholesterol ester transformation, was stained by IF (n = 2). Decreased expression and altered distribution could be detected. All images (including the secondary antibody control) were taken with the same exposure times. (f-h) As a proof-of-concept experiment, HAoEC were incubated with CcA to inhibit the vATPase and thereby neutralize lysosomal pH, or CI-976 to inhibit SOAT-1, or their combination in presence of LDL. (f) PLM analysis revealed strongly increased LDL-derived CC production when lysosomal pH was increased in the presence of SOAT-1 inhibition (each n = 9). (d) SEM reveals increased size and presence of observed CC (n = 2). (e) TEM shows development of ‘clefts’ (asterix) similar to LDL and LDL+ IFNγ/ TNFα treated HAoEC. Observed ‘clefts’ are indicative for CC presence. (Abbreviations: CcA-concanamycin, CC-cholesterol crystal, L-lumen, PD-petri dish, psEC-CC – possible EC-derived CC, Statistics: Friedman ANOVA test; *indicates significance to LDL)

**Fig. 5:**
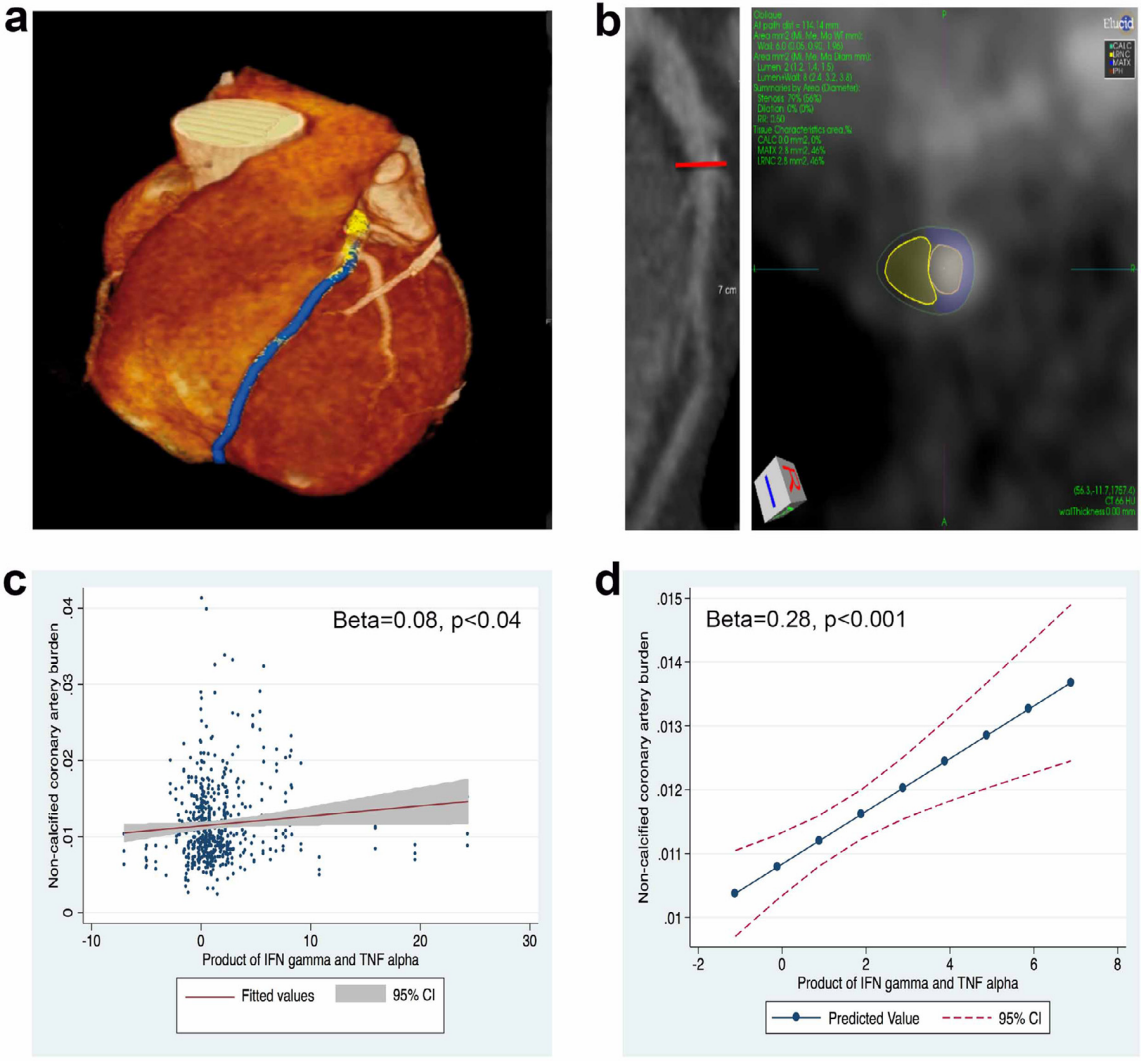
Synergism between IFNγ/TNFα is associated with early non-calcified coronary burden in human psoriasis. a) 3D reconstruction of the heart highlighting the left anterior descending coronary artery acquired from the Coronary Computed Tomography Angiography scan. b) A curved planar reconstruction image of longitudinal LAD with the red line corresponding to tomographic view with color overlay demonstrating an early lipid-rich coronary plaque subcomponent in yellow. c) Unadjusted association between IFNγ/TNFα product and early lipid-rich non-calcified coronary artery burden along with corresponding scatter representing data points. d) Adjusted association between IFNγ/TNFα product and non-calcified coronary artery burden along with corresponding fitted line beyond adjustment for age, sex, hypertension, hyperlipidemia, waist: hip ratio, statin use as well as individual IFNγ and TNFα levels. The standardized *β*-values along with the corresponding p-value is indicated in the graph.

Finally, we focused on the cholesterol balance within HAoEC under the given treatment conditions (Fig. 4). We determined that LDL+IFNγ/TNFα synergistically increased free cholesterol load compared to the individual treatments, while no differences were observed for neutral lipid /cholesterol ester load (Fig. 4a/b). Overall lipid uptake over 24h was highest in the LDL+IFNγ/TNFα conditions (Fig. 4c). In a last step, we aimed to identify if cholesterol uptake or sensing mechanisms might be altered in LDL+IFNγ/TNFα treated HAoEC (Fig. 4d). For this, HAoEC were treated as indicated for 24 h, followed by 2 h uptake of DiI-labelled LDL detection. LDL, nor LDL+IFNγ pre-treated cells, took up further DiI-LDL when compared to non-lipid or cytokine treated controls. However, LDL+TNFα treated cells displayed continuous lipid uptake with no additional increase by addition of IFNγ, potentially being the main contributor to the 24 h uptake results in Fig. 5c. The regulation of cellular free and esterified cholesterol content is regulated by various proteins including SOAT-1 which converts free cholesterol to cholesterol esters and has been implicated in CC formation.[28,38] IF staining of SOAT-1 revealed decreased expression when HAoEC were treated with LDL+IFNγ/TNFα (Fig. 4e).

Finally, we undertook a second proof-of-principle experiment and combined concanamycin (neutralizing lysosomal pH) with CI-976 (SOAT-1 inhibition) in presence of LDL to determine if both mechanisms potentially need to be impaired to accelerate EC-derived CC formation as determined by PLM (Fig. 4f). SOAT-1 inhibition by CI-976 (+LDL) increased the presence and size of CC (1.6 ± 0.25-fold change). Again, concanamycin did not increase LDL-induced CC presence (0.99 ± 0.1-fold change). However, the combination of both substances, CI-976/CcA, accelerated LDL-induced CC production by 3.8 ± 0.7 (fold change). CC produced by HAoEC after CI-976/CcA + LDL treatment were visible in SEM (Fig. 4g) and displayed ‘cleft’ formation within HAoEC (Fig. 4h).

### Synergism between IFNγ/TNFα is associated with early non-calcified coronary burden in human psoriasis

After determining a combined effect of these cytokines on endothelial CC formation, we tested a possible relevance in human CVD. Consecutive 224 patients with psoriasis underwent coronary computed tomography angiography scans to assess coronary arterycharacteristics of total coronary burden (TB) and non-calcified coronary burden (NCB). The psoriasis cohort (summarized in Table 1, Supplementary Fig. 4) was middle aged, predominantly male, low cardiovascular risk by Framingham risk score and moderate-to-severe psoriasis. Neither IFNγ *(β* *=* *0.01, p* *=* *0.71*) nor TNFα *(β* *=* *0.07, p* *=* *0.06*) reached statistical significance in their individual associations with NCB (fully adjusted model included age, sex, hypertension, hyperlipidaemia, waist: hip ratio and statin use). However, the product of IFNγ and TNFα positively associated with NCB in models adjusted for age, sex, hypertension, hyperlipidaemia, waist: hip ratio, statin use as well as individual IFNγ and TNFα levels (*β* *=* *0.28, p* *<* *0.001*; Fig. 5). Similar relationship was seen with TB (adjusted: *β = 0.25, p = 0.001).*

**Table 1:** Summary of psoriasis participants.

Variable	All
	(N = 224)
Age, years	50.0 ± 12.3
Males	133 (59)
Hypertension	67 (30)
Hyperlipidaemia	93 (42)
Type-2 diabetes	22 (10)
Waist-to-hip ratio	0.95 (0.90–1.00)
Current smoker	28 (12.5)
Statin use	66 (29.5)
Total cholesterol, mg/dL	185.3 ± 37.6
HDL cholesterol, mg/dL	56.7 ± 17.2
LDL cholesterol, mg/dL	105.3 ± 31.0
Triglycerides, mg/dL	119.5 ± 65.8
Framingham risk score	2 (1–6)
C-reactive protein, mg/L	2 (1–5)
Psoriasis area severity index score	6 (3–10)
Total body surface area index	2 (1–5)
Biologic psoriasis treatment	60 (27)
IFNγ, pg/mL	20.78 ± 55.73
TNFα, pg/mL	4.01 ± 7.68

Values reported in the table as Mean ± SD or Median (IQR) for continuous data and N (%) for categorical data. IFNγ-interferon gamma. TNFα-tumor necrosis factor alpha.

## Discussion

Our work demonstrates a role of IFNγ/TNFα in driving CC formation due to modulation of lysosomal pH and an increase of intracellular free cholesterol load. Our cell culture experiments suggest a link between lysosomal pH neutralization and increased free cholesterol load to CC formation under pro-inflammatory and hyperlipidaemic conditions potentially due to decreased presence and altered distribution of vATPaseV1E1 and SOAT-1. Furthermore, SOAT-1 reduction suggests that cholesterol imbalance in the presence of inflammation drives the formation of CC in ECs. Finally, *in vivo* we found that plasma levels of these critical inflammatory cytokines synergistically associate with early, lipid-rich non-calcified coronary burden. Taken together, these findings provide early mechanistic links of the role of chronic inflammatory dyslipidaemia in modulating endothelial cell biology and promote early atherosclerosis through formation of CCs.

We found the presences of CCs in a mouse model of chronic skin inflammation on chow diet resembling phenotypic characteristics of changes observed in an atherosclerotic mouse model after 2 weeks of HFD. The striking similarities between 2-week HFD and psoriatic mouse aortas without HFD being fed supported the idea of inflammation-accelerated deposition of CC and lipids in the subendothelial space. The fact that these ultrastructural similarities could be observed without HFD is striking since we reported previously that this psoriasis mouse model does not display overt hyperlipidaemia yet a dysfunctional lipid profile.[30] Furthermore, the ultrastructural analysis displayed subendothelial spaces and ‘clefts’ generally believed to be sign of crystal presence in TEM. These morphological changes, the subendothelial deposition of lipids and early CC, have been described to be one of the first pathological changes in the arterial wall during atherogenesis.[18,28,39,40] Therefore, chronic inflammation and lipid dysfunction in psoriasis may accelerate CC formation and atherogenesis in psoriasis-related CVD.

Second, we found important effects of cytokines known to be active in psoriasis, specifically IFNγ and TNFα; a finding which was supported by a prior report of synergism of these cytokines in psoriasis skin and human atherosclerotic plaques.[32] Other reports have demonstrated various cellular mechanisms in various cell types modulated by these two cytokines.^41^, ^42^, ^43^, ^44^, ^45^

Of particular interest was that we determined an increase in lysosomal pH under hyperlipidaemic conditions in the presence of IFNγ and TNFα. While earlier reports have demonstrated presence of CC in lysosomal structures within macrophages after oxLDL treatment [27,29], the link between intracellular CC formation and lysosomal pH has not been reported previously. Our finding is furthermore substantiated by experiments which showed that chemically defined CC formation resulted in increased CC volumes when pH was altered from acidic to basic conditions.[35] Because CC have been studied in the context of acute treatment of cells[19,20,46,47], the circumstances of CC formation or pathways in chronic conditions remain understudied. This interplay and dysregulation in the setting of chronic inflammatory diseases should be further investigated to further implicate lysosomal function accompained lipid metabolism dysregulation in inflammatory CVD.

Moreover, we also found a modulation of a vATPase subunit V1E1 localization. The lysosomal vATPase is an essential regulator of lysosomal pH. Interestingly in support of our findings where subunits seem to be dysregulated, the V0A1 subunit has been described as ‘misrouted’ in a model of neurodegenerative lysosomal storage diseases, specifically by altering lysosomal acidification.[37] The importance of inflammation and lysosomal pH in a setting outside of lipid metabolism and atherogenesis has been recently demonstrated for lupus. Macrophages of a lupus-prone mouse model displayed a chronic activation of mTORC2 which has been directly linked to lysosomal acidification subsequently leading to multiple lupus-associated pathologies.[48]

In addition to dysregulated lysosomal pH, we also detected that EC lipid homeostasis was altered by LDL+IFNγ/TNFα treatment as indicated by a synergistically increased free cholesterol load. We found that SOAT-1 presence was decreased which could at least partially explain why intracellular levels of free cholesterol were significantly increased in the treated HAoEC. Interestingly, *SOAT*^*−/−*^*/ApoE*^*−/−*^ mice display skin xanthomatosis filled with large amounts of CC when on chow diet which indicates that a lack of SOAT-1 under normolipidemic conditions gives rise to CC.[38] This is in accordance to our findings where we suspect SOAT-1 to be less expressed along with increased CC formation by ECs. Chronic inflammation, as present in psoriasis, may alter SOAT-1 and under normolipidemic conditions give rise to CC leading to accelerated atherogenesis under hyperlipidaemic conditions. Since SOAT-1 inhibitors have not resulted in decrease in CVD in the ACTIVATE and CAPTIVATE trials [49,50], it may be that CC production was not inhibited

Psoriasis is a chronic inflammatory skin disease associated with elevated systemic inflammation, early dyslipidaemia and increased subclinical as well as clinical atherosclerosis in the form of lipid-rich non-calcified coronary disease by coronary computed tomography [7,8,51] and incident myocardial infarction.[5,52, 53, 54, 55, 56, 57, 58, 59] In fact, the elevated cardiovascular risk seen in psoriasis patients is in part due to dysregulation of superimposed lipid and inflammatory pathways. Therefore, psoriasis provides a model to characterize associations between inflammation, dyslipidemia and early coronary artery disease before cardiovascular events.[55,60, 61, 62, 63, 64] In that context, our study demonstrated that the product of chronic inflammatory cytokines in the form of IFNγ/TNFα associated with non-calcified coronary burden beyond traditional risk factors. However, these findings appear to be driven by TNFα. This is not unanticipated since TNFα is the dominant cytokine in the pathogenesis of cutaneous, joint, vascular and metabolic manifestations of psoriasis.[59] In fact, anti-TNFα agents are the mainstay and longest standing anti-inflammatory biological class of drugs approved to treat skin disease in psoriasis.[65] Indeed, psoriasis patients receiving anti-TNFα agents have showed reduction in clinical cardiovascular disease including myocardial infarction as well as sub-clinical cardiovascular disease in the form of non-calcified coronary disease and peri-coronary inflammation over time.[8,66, 67, 68]

CC are often used in the experimental setting of an acute treatment of cells and a major finding has been the activation of the NLRP3 inflammasome including a lysosome-related pathway and its subsequent impact on atherosclerosis development and progression.[20,69] However, we were unable to detect activation of the NLRP3 inflammasome in our study. We recognize that the use of the product of two cytokines in our experiments is only a crude measurement of true cytokine synergy. However, we hope that the presentation of the results of individual as well as combined treatment support the idea of synergistic function. We also recognize that more studies are needed involving knockout mouse models, to further understand the mechanisms underlying the observed pH changes and alterations in lipid homeostasis within EC.

In summary, our observations provide early evidence that inflammatory CVD associates with lipid-derived crystals and lipid rich coronary burden. Future work should determine the impact of inflammation on regulators of lysosomal function and lipid homeostasis under normo- or hyperlipidaemic conditions. These processes should be investigated in parallel as we found evidence that inflammatory induced lysosomal dysfunction and intracellular lipid homeostasis seem to be tightly connected especially since a recent study demonstrated vATPases to be of crucial importance for ABCA1-mediated HDL efflux [70], a process known to be impaired in psoriasis patients as well.[12] Future therapeutic strategies could address lysosomal re-acidification as a mechanism by which to ultimately decrease CVD risk in chronically inflamed patients.

## Author Contributions

YB and NNM designed the study. YB, AKD, and NNM drafted the manuscript. YB performed most of the experiments. GES and QN took care of the animals. PKD and AS helped with all flow cytometry related experiments. ES and CKEB helped with the TEM experiments. YS performed the transfections experiment and imaging involving pH sensitive probe. AKD, CAG-H, YB, NOK, and YAE performed statistical analysis of experiments involving human specimens. Images from *Ldlr^−/−^* mice were provided by WAB from experiments performed by YB under WAB supervision. MYC and DAB were involved in acquisition of CCTA data. AK and JAR were involved in patient recruitment and specimen handling. MPP, TMPW and NNM supervised the work. WAB, HSK, DMS, HLT, LCT, JEG, DMS and JMG as well as all other authors provided critical revisions of the manuscript.

## Data availability

The dataset generated and analysed during the current study are available from the corresponding author on reasonable request.

## Declaration of Interest

Dr. Mehta has received funding from the National Institutes of Health Intramural Research Program (Z01 HL-06193); and has received research grants from Abbvie, Janssen, Novartis Corp, and Celgene. Dr. Gelfand reports personal fees from BMS, Boehringer Ingelheim, GSK, Lilly, Janssen Biologics, Novartis Corp., UCB (DSMB), Neuroder (DSMB), Dr. Reddy's Labs, Pfizer Inc., and Sun Pharma as well as grants from Abbvie, Boehringer Ingelheim, Janssen, Novartis Corp., Celgene, Ortho Dermatologics, and Pfizer, outside the submitted work. All other authors have reported that they have no relationships relevant to the contents of this paper to disclose.

## Appendix Supplementary materials

Image, application 1

## Footnotes

Supplementary material associated with this article can be found, in the online version, at doi:10.1016/j.ebiom.2020.102876.
