# Supplementary material for: Hyperlipidaemia and IFNgamma/TNFalpha Synergism are associated with cholesterol crystal formation in Endothelial cells partly through modulation of Lysosomal pH and Cholesterol homeostasis
Source: eBioMedicine. 2020 Jul 6;59:102876. doi: 10.1016/j.ebiom.2020.102876 (PMC7502673; doi:10.1016/j.ebiom.2020.102876)
Supplement: Supplementary file 1 [file mmc1.pdf]

ONLINE SUPPLEMENT

Supplementary Figures and Figure Legends

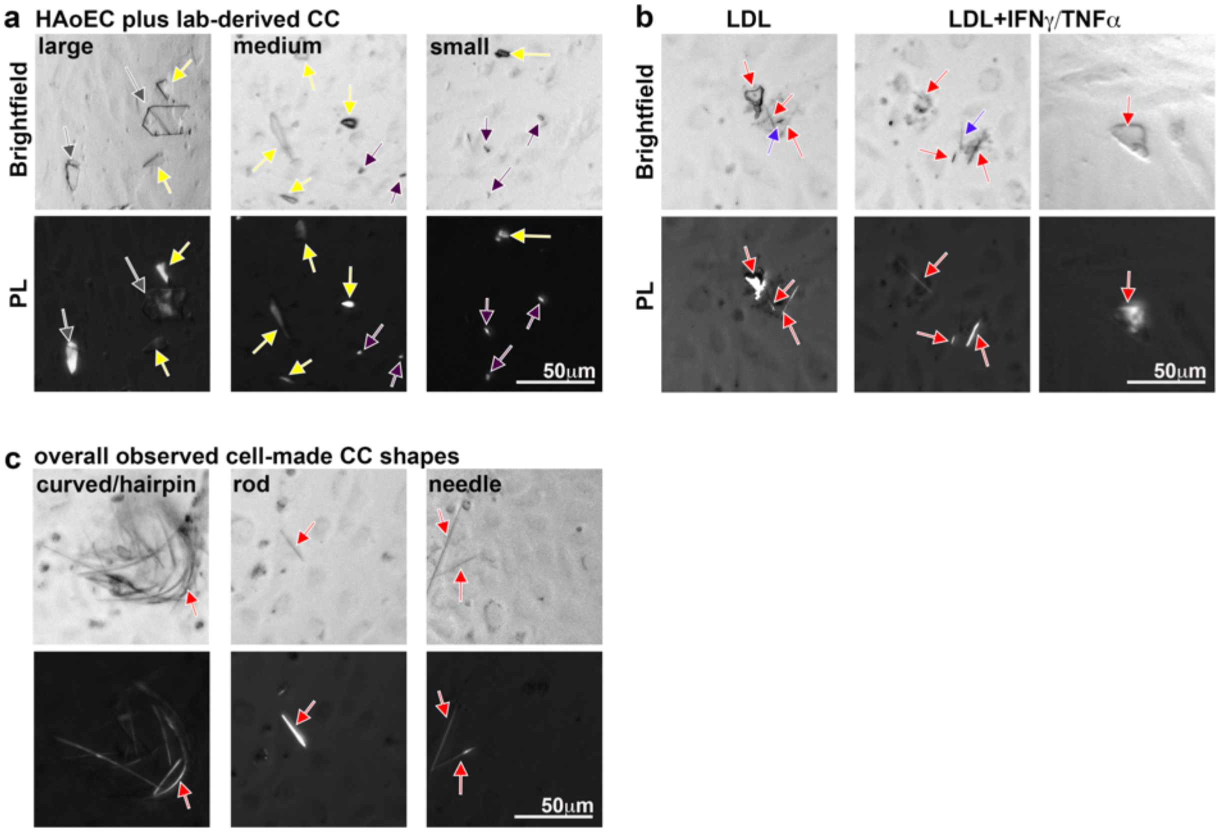

Supplementary Figure 1. Internal assay controls confirm appearance of CC in PLM to be similar to CC derived by HAoEC. HAoEC were treated as indicated. (**a**) Brightfield and PLM images of HAoEC treated with lab-derived CC are shown. Various sizes of lab-derived crystals can be visualized. (large-grey, medium-yellow, small-purple). (**b/c**) Endothelial derived CC appear in various shapes and sizes as shown in PLM and brightfield images.

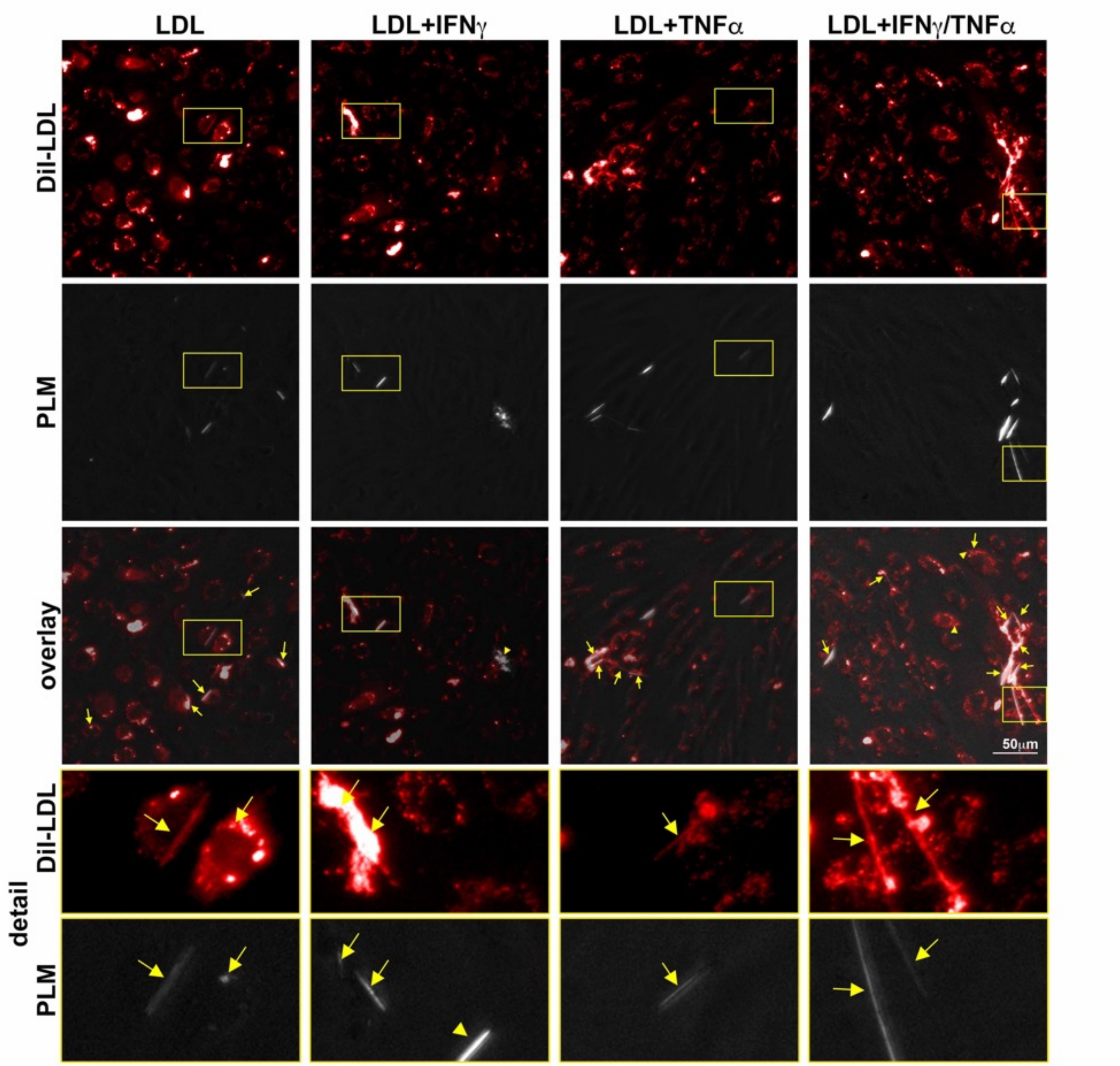

Supplementary Figure 2. Observed endothelial cell-produced CC are LDL-derived. HAoEC were treated with DiI-labelled LDL and the addition of cytokines as indicated for 24h. After fixation, HAoEC were simultaneously imaged for DiI-fluorescence in PLM to determine if observed bifringent structures (CC) are DiI-positive which indicates their origin to be DiI-labelled LDL. We found an increase in elongated rod-like structures (arrow heads) as well as increased presence of large DiI-positive accumulations/vesicles (arrows) when treated in combination with IFNγ/ TNFα. (n=2). (yellow boxes indicate the area shown in the detail at higher magnification)

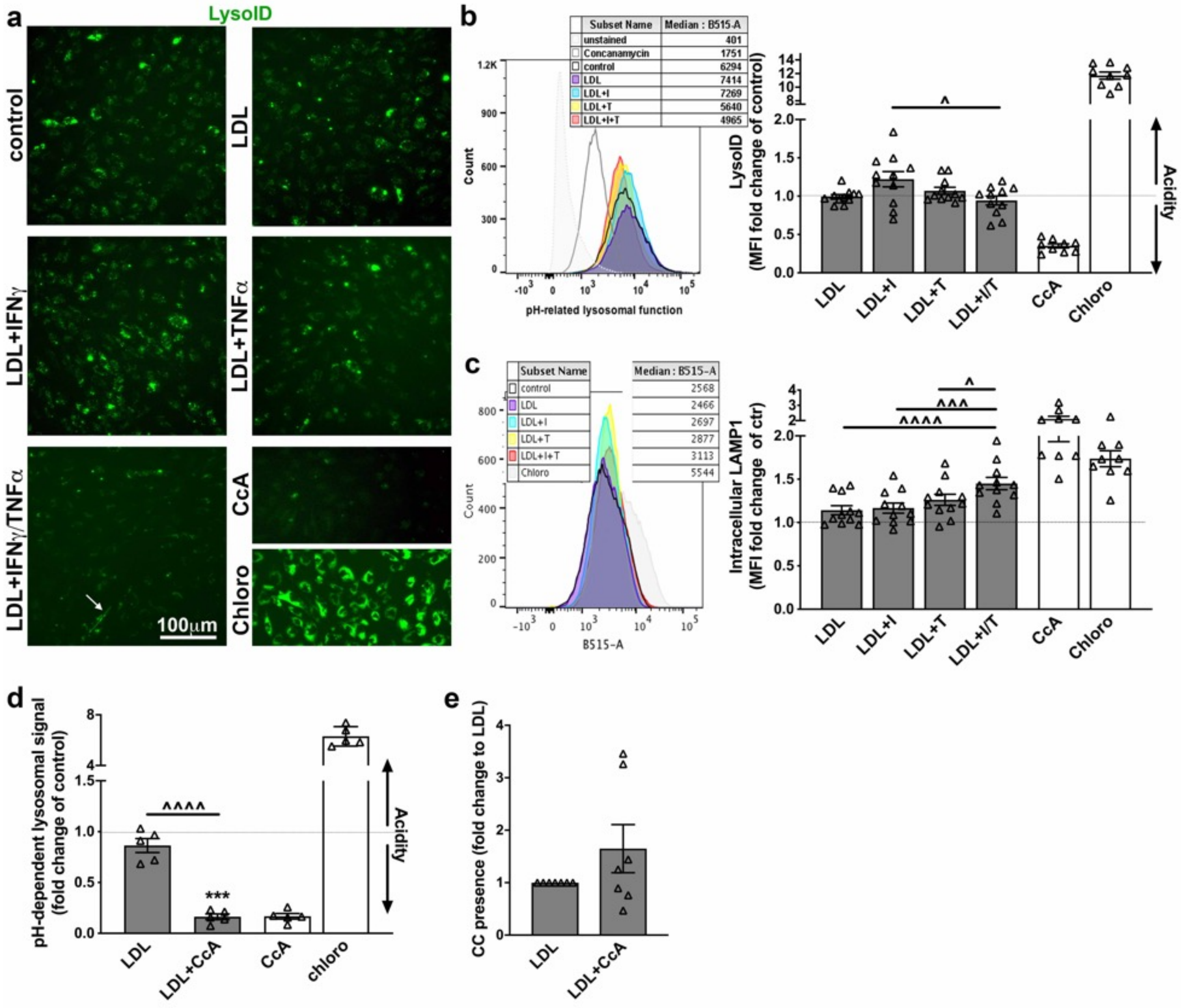

Supplementary Figure 3. CC formation in HAoEC is accompanied by changes in the endo-lysosomal pathway. (**a**/**b**) HAoEC were treated as indicated for 24h, incubated with LysoID, an indicator of lysosomal pH, and subsequently examined by fluorescence microscopy and flow cytometry. LysoID emits a green fluorescence upon entering lysosomes with its intensity being dependent on pH and therefore indicating lysosomal pH changes. Increasing/neutralizing pH will decrease the emitted green signal. Concanamycin (neutralizes lysosomal pH=decreases signal) and chloroquine (acidifies lysosomes=increases signal) were used as internal assay controls. (**a**) Fluorescence images were taken with equal exposure times and LDL+ IFNγ/TNFα treatment resulted in a clear decrease of detectable green fluorescence. (n=3) (**b**) A representative Histogram of the MFI detected by flow cytometry is shown with the results of n=11 summarized in the graph on the right. (**c**) In parallel the presence of intracellular LAMP1 was determined using flow cytometry. (n=11) (Statistics: b/c-Friedman test) (**d**/**e**) In a proof-of-concept experiment HAoEC were treated with Concanamycin (CcA) in presence of LDL. A loss in pH-dependent lysosomal signal, meaning an increase in lysosomal pH, was verified for this experimental design (n=5, RM-one-way ANOVA test). CC formation was detected using PLM (**f**) (n=7, paired t-test). (Statistics: *significance to control, ^significance between indicated groups)

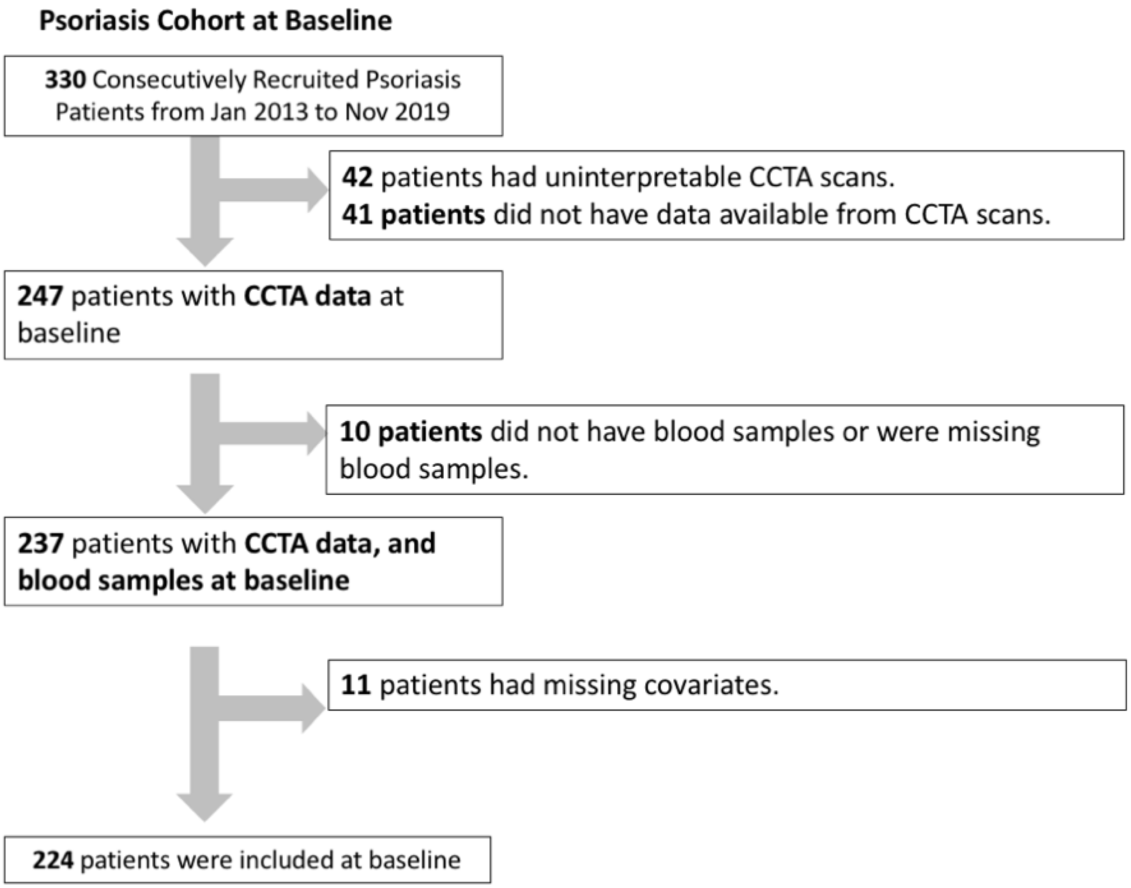

Supplementary Figure 4. Recruitment schematic of 224 psoriasis patients included in the study.

Supplementary Tables

Supplementary Table 1: Key Resource Table summarizing materials, kits, and antibodies used in this study.

REAGENT or RESOURCE	SOURCE	IDENTIFIER
**Antibodies and Labelling Substances**		
GAPDH Monoclonal Antibody (6C5)	Thermo Fisher Scientific Cat# AM4300	RRID:AB_2536381
LAMP1 Monoclonal Antibody (H4A3), Alexa Fluor 488	Thermo Fisher Scientific Cat# MA5-18121	RRID:AB_2539495
LAMP1 Monoclonal Antibody (H4A3), FITC	Thermo Fisher Scientific Cat# A14750	RRID:AB_2534266
vATPase V1E1	Thermo Fisher Cat# PA-529899	RRID:AB_2547373
SOAT-11	Novus Biologicals	Cat# NBP2-32052
Alexa Fluor ™ 488 Phalloidin	Thermo Fisher	Cat# A12379
Goat anti-rabbit cy3	AbCAM	Cat# ab6939
**Chemicals, Peptides, and Recombinant Proteins**		
Recombinant Human IFNγ	BD Biosciences	Cat# 554617
Recombinant Human IFNγ	PeproTech	Cat# 300-02
Recombinant Human TNFα	BD Biosciences	Cat# 554618
Recombinant Human TNFα	PeproTech	Cat# 300-01A
Recombinant Human IL-1β	PeproTech	Cat# 200-01B
Recombinant Human IL-6	PeproTech	Cat# 200-06
Recombinant Human IL-8	PeproTech	Cat# 200-08
Recombinant Human IL-17	BioLegend	Cat# 574102
Concanamycin	Tocris	Cat# 2656
Chloroquine	Tocris	Cat# 4109
CI-976	Tocris	Cat# 2227
Cholesterol	Sigma Aldrich	C8667-5g
LIPOPROTEIN LO DENSITY 99 5MG, Alfa Aesar	VWR	AAJ65039-LB0
Dii-Lipoprotein Lo D 200Ug, Alfa Aeser	VWR	AAJ65330-AMH
Low density lipoprotein	Millipore EMD	437644-10mg
Filipin complex from Streptomyces filipinensis	Sigma Aldrich	F9765-50MG
HCS LipidTOX™ Deep Red Neutral Lipid Stain, for cellular imaging	ThermoFisher Scientific	H34477
**Critical Commercial Assays**		
LYSO-ID® Green detection kit	Enzo Life Sciences	ENZ-51034-K500
LysoTracker™ Blue DND-22	ThermoFisher Scientific	L7525
**Experimental Models: Cell Lines**		

Primary Human Aortic Endothelial Cells	PromoCell, Germany	C-12272
**Experimental Models: Organisms/Strains**		
K14-Rac1V12 mouse model	Marinkovich Lab *J Clin Invest.* 2016;126(7):2661–2677. doi:10.1172/JCI85738	
**Recombinant DNA**		
pCMV-lyso-pHoenix	Addgene #70112	RRID:Addgene_70112
**Software and Algorithms**		
Stata 12	StataCorp., College Station, TX, USA	RRID:SCR_012763
GraphPad PRISM 7	GraphPad Software, Inc., San Diego, CA	RRID:SCR_002798
FlowJo software version 9 and 10	FlowJo9/10, LLC, Ashland, Ore	RRID:SCR_008520
